# Angiotensin-(1–7) suppresses airway inflammation and airway remodeling via inhibiting ATG5 in allergic asthma

**DOI:** 10.1186/s12890-023-02719-7

**Published:** 2023-11-02

**Authors:** Jianfeng Xu, Zhenyu Yu, Xueping Liu

**Affiliations:** 1 Department of Pulmonary and Critical Care Medicine, Yantai Yuhuangding Hospital, No.20, Yuhuangding East Road, Zhifu District, Yantai, 264001 China; 2 Department of Anesthesiology, Yantai Yuhuangding Hospital, Yantai, 246001 China

**Keywords:** Transforming growth factor (TGF)-β1, Type 2 helper T cell (Th2) cytokines, Autophagy related (ATG)-5, ATG5 knockout (ATG5^−/−^) mice

## Abstract

**Background:**

Angiotensin (Ang)-(1–7) can reduce airway inflammation and airway remodeling in allergic asthma. Autophagy-related 5 (ATG5) has attracted wide attentions in asthma. However, the effects of Ang-(1–7) on ATG5-mediated autophagy in allergic asthma are unclear.

**Methods:**

In this study, human bronchial epithelial cell (BEAS-2B) and human bronchial smooth muscle cell (HBSMC) were treated with different dose of Ang-(1–7) to observe changes of cell viability. Changes of ATG5 protein expression were measured in 10 ng/mL of interleukin (IL)-13-treated cells. Transfection of ATG5 small interference RNA (siRNA) or ATG5 cDNA in cells was used to analyze the effects of ATG5 on secretion of cytokines in the IL-13-treated cells. The effects of Ang-(1–7) were compared to the effects of ATG5 siRNA transfection or ATG5 cDNA transfection in the IL-13-treated cells. In wild-type (WT) mice and ATG5 knockout (ATG5^−/−^) mice, ovalbumin (OVA)-induced airway inflammation, fibrosis and autophagy were observed. In the OVA-induced WT mice, Ang-(1–7) treatment was performed to observe its effects on airway inflammation, fibrosis and autophagy.

**Results:**

The results showed that ATG5 protein level was decreased with Ang-(1–7) dose administration in the IL-13-treated BEAS-2B and IL13-treated HBSMC. Ang-(1–7) played similar results to ATG5 siRNA that it suppressed the secretion of IL-25 and IL-13 in the IL-13-treated BEAS-2B cells, and inhibited the expression of transforming growth factor (TGF)-β1 and α-smooth muscle actin (α-SMA) protein in the IL-13-treated HBSMC cells. ATG5 cDNA treatment significantly increased the secretion of IL-25 and IL-13 and expression of TGF-β1 and α-SMA protein in IL-13-treated cells. Ang-(1–7) treatment suppressed the effects of ATG5 cDNA in the IL-13-treated cells. In OVA-induced WT mice, Ang-(1–7) treatment suppressed airway inflammation, remodeling and autophagy. ATG5 knockout also suppressed the airway inflammation, remodeling and autophagy.

**Conclusions:**

Ang-(1–7) treatment suppressed airway inflammation and remodeling in allergic asthma through inhibiting ATG5, providing an underlying mechanism of Ang-(1–7) for allergic asthma treatment.

**Supplementary Information:**

The online version contains supplementary material available at 10.1186/s12890-023-02719-7.

## Introduction

Allergic asthma, a type of bronchial asthma, is driven by the type 2 helper T cell (Th2) immune response of inhaled air allergens, characterizing airway hyperresponsiveness (AHR), production of Th2 cytokines, and structural changes in the airway wall [1–3]. Upon exposure to an allergen, airway epithelial cells secrete interleukin (IL)-25 and IL-33 to initiate Th2 immune response and induce the secretion of Th2 cytokines, IL-4, IL-5 and IL-13 [3]. The secretion of Th2 cytokines further affects airway remodeling through inducing aggregation of eosinophils, neutrophils, lymphocytes and macrophages. Airway remodeling is characterized by matrix deposition and enhanced smooth muscle mass in the airways. Smooth muscle cells secrete transforming growth factor (TGF)-β1, collagens and α-smooth muscle actin (α-SMA) to promote airway remodeling in asthma [4, 5]. In clinical practice, combination of corticosteroids and long-term effect β2 adrenergic receptor agonists is commonly used as the main treatment method [6]. However, long-term or high-dose use can lead to steroid insensitivity and β2-adrenoceptor-desensitization. Further identification of key molecules or related genes in allergic asthma may provide greater insight into developing novel treatments, which may overcome the limitations of current treatments. Therefore, further research on key molecules or related genes in allergic asthma is of great significance for developing therapeutic strategies.

Studies suggest that mitochondrial dysfunction is key to the pathogenesis of allergic asthma [7–9]. Autophagy is an intracellular process in which damaged organelle are cleared and recycled. Disruptions of normal autophagy processes can lead to the accumulation of damaged organelle (such as mitochondria), and releases reactive oxygen species (ROS), thus affecting cell apoptosis [10]. Accumulating evidence have shown that autophagy affects cellular immune responses, especially the survival and activation of T lymphocytes [7, 8]. In allergic asthma, autophagy is activated and autophagy related proteins, autophagy related (ATG)-5, Beclin-1, light chain 3B (LC3B) and p62, are overexpressed in the tissues of asthma patients [8, 11]. ATG5 represents a key protein involved in the formation of autophagosomes and LC3B [12]. Inhibiting ATG5-mediated autophagy can reduce airway inflammation and AHR in asthma [13].

Angiotensin (Ang)-(1–7) is a 7-peptide composed of aspartic acid, arginine, valine, tyrosine, isoleucine, histidine and proline, and acts in the nonclassical renin-angiotensin system (RAS) through the Mas receptor [14]. The Ang-(1–7)/Mas receptor axis plays protective roles in various tissues. Moreover, Mas receptor expression decreases in acute allergic airway inflammation, which regulates chemokine (C–C motif) ligand 2 (CCL-2)-mediated macrophage infiltration through JNK pathways in airway inflammation [15]. Ang-(1–7) treatment can inhibit airway inflammation and reduce airway remodeling in allergic asthma [14–17]. Inhalation of Ang-(1–7) can reduce OVA-induced inflammation and collagen deposition through reducing eosinophils, expression of IL-5, CCL11, IgE, metalloproteinase (MMP)-9 and MMP-12 in mice [14]. In cigarette smoking-induced pulmonary fibrosis, the autophagy-related proteins, LC3B and P62, are significantly increased, and Ang-(1–7) treatment improves the impaired autophagy via reducing the nicotinamide adenine dinucleotide phosphate reduced oxidase (NOX) 4-mediated ROS [18]. However, it is unclear whether the anti-inflammatory mechanism of Ang-(1–7) in allergic asthma is related to ATG5-mediated autophagy.

In this study, IL-13-treated human normal bronchial epithelial cells (BEAS-2B) and IL-13-treated human bronchial smooth muscle cells (HBSMC) were used to analyze the effects of Ang-(1–7) on ATG5-mediated autophagy. In vivo, ovalbumin (OVA)-induced wild type (WT) mice were used to analyze the roles of Ang-(1–7) on autophagy. Meanwhile, the ATG5 knockout (ATG5^−/−^) mice were used to confirm the effects of ATG5 in allergic asthma.

## Materials and methods

### Cell culture

Human bronchial epithelial cell (BEAS-2B, #CL-0496) and human bronchial smooth muscle cell (HBSMC, #CP-H012) were purchased from Procell (https://www.procell.com.cn/, Wuhan, China) and cultured in a Dulbecco’s Modified Eagle Medium (DMEM) containing 10% fetal bovine serum (FBS) and 1% penicillin and streptomycin at 37 °C with 5% CO_2_. The same cell passage (P3) was used in all experiments to avoid changes of cell viability. Cells at 60–70% confluence were used for experiments.

### Effects of Ang-(1–7) on cell viability

BEAS-2B and HBSMC cells (1 × 10^7^ cells/well) were cultured in 96-well plates and treated with different doses (5, 10, 20, and 40 µM) of Ang-(1–7) for 24 h at 37 °C with 5% CO_2_. The Ang-(1–7) (#HY-12403, MedChemExpress, China) was dissolved in 1% phosphate buffer solution (PBS), and 1% PBS was used as a control. Cell viability was measured using a cell counting kit (CCK)-8 kit (#CA1210-1000 T, Solarbio® Life Science, Beijing, China).

### Effects of Ang-(1–7) on ATG5 protein expression in IL-13-treated cells

BEAS-2B and HBSMC cells were treated with 10 ng/mL human IL-13 (#HY-P72795, MedChemExpress, China) for 24 h, and then treated with different doses (5, 10, and 20 µM) of Ang-(1–7) for 24 h at 37 °C with 5% CO_2_. IL-13 was dissolved in 1% PBS, and 1% PBS was used as a control. Expression of ATG5 protein was measured by western blotting.

### Cell transfection

According to previously reported methods [19, 20], the BEAS-2B and HBSMC cells were transfected with 20 nM of human control small interference RNA (siRNA, 5ʹ-UUCUCCGAACGUGUCACGA-3ʹ) or ATG5 siRNA (5ʹ-CATCTGAGCTACCCGGATA-3ʹ) using Lipofectamine 2000 reagent for 48 h. The products of control siRNA or ATG5 siRNA were purchased from GenePharma (http://www.genepharma.com/en/, Shanghai, China). The results of ATG5 siRNA transfection were confirmed by western blotting (Supplementary Fig. 1).

As previously reported [21, 22], the BEAS-2B and HBSMC cells were transfected with adenovirus vector encoding human ATG5 cDNA or vector control (GenePharma, Shanghai, China) for 24 h using Liposome 8000 reagent. ATG5 overexpression adenovirus were obtained through cloning the cDNA of ATG5 gene (Forward, 5′-GTCAGATCCGCTAGAGATCTGCTTACTAAGTTTGGCTTTGGTT-3′ and reverse, 5′-GATATCTTATCTAGAAGCTTAAGGGTGACATGCTCTGATAAAT-3′) into the pAdTrack-CMV. The results of ATG5 cDNA transfection were confirmed by western blotting (Supplementary Fig. 2).

### Cell groups

First, the effects of 20 µM Ang-(1–7) treatment alone on secretion of cytokines were observed. The BEAS-2B and HBSMC cells were respectively divided into control group, 1% PBS group and Ang-(1–7) group. The cells were treated with 1% PBS or 20 µM Ang-(1–7) for 24 h.

The effects of Ang-(1–7) treatment in the IL-13-treated cells were compared with the results of ATG5 siRNA transfection in the IL-13-treated cells. The BEAS-2B and HBSMC cells were respectively divided into five groups: control group, IL-13 + control siRNA group, IL-13 + ATG5 siRNA group, IL-13 + Ang-(1–7) group, and IL-13 + PBS group. In the IL-13 + control siRNA group or IL-13 + ATG5 siRNA group, cells were transfected with control siRNA or ATG5 siRNA, then treated with 10 ng/mL IL-13 for 24 h. In the IL-13 + Ang-(1–7) group, cells were treated with 10 ng/mL IL-13 for 24 h, then treated with 20 µM Ang-(1–7) for 24 h.

Furthermore, the ATG5 overexpressing cells were used to analyze a mechanism of Ang-(1–7) in IL-13-treated cells. The BEAS-2B and HBSMC cells were respectively divided into five group: control group, ATG5 cDNA + IL-13 group, vector control + IL-13 group, ATG5 + IL-13 + Ang-(1–7) group, and IL-13 + vector + Ang-(1–7) group. In the ATG5 cDNA + IL-13 group or vector control + IL-13 group, cells were transfected with adenovirus vector encoding human ATG5 cDNA or vector control, then treated with 10 ng/mL IL-13 for 24 h. In the ATG5 + IL-13 + Ang-(1–7) group, the ATG5 cDNA-transected cells were treated with 10 ng/mL IL-13 for 24 h, then treated with 20 µM Ang-(1–7) for 24 h.

### Western blotting

According to previously reported method [23], total proteins were isolated from cells or tissues, separated with sodium dodecyl sulfate–polyacrylamide gel electrophoresis and transferred to a polyvinylidene fluoride membrane. Prior to incubated with antibodies, the membranes were cut and then incubated with anti-rabbit ATG5 antibody (1:1000, #A0203, ABclonal, China), anti-rabbit Beclin-1 antibody (1:1000, #A7353, ABclonal), anti-rabbit transforming growth factor (TGF)-β1 antibody (1:1000, #A7040, ABclonal), anti-rabbit α- smooth muscle actin (SMA) antibody (1:1000, #A17910, ABclonal) and anti-rabbit GAPDH antibody (1:1000, #AC001, ABclonal) overnight at 4 °C. After washing, the membrane was cultured with the second antibody (1:1000, AS029, ABclonal) for 60 min at 37 °C. An enhanced chemiluminescence reagent kit (#K1230, ApexBio, Shanghai, China) was used to visualize the bands.

### Enzyme linked immunosorbent assay (ELISA)

Levels of IL-25 (#ab272200, Abcam, China) and IL-33 (#PI631, Beyotime, Shanghai China) in supernatants of BEAS-2B cells were measured using ELISA kits. The supernatants were collected through centrifugation (× 800 g) for 10 min.

### Animals

Fifteen of 6–8 weeks old wild-type (WT) C57BL/6 J male mice (Jinan Pengyue Experimental Animal Breeding Co., Ltd., China) and ten of 6–8 weeks old ATG5^−/−^ C57BL/6 J male mice (C57BL/6 J-Atg5^em1cyagen^, Cyagen, China) were housed under specific pathogen free conditions for experiments.

### Animal model

Allergic asthma model of mice was induced as previous reports [3, 24]. In a short, mice were sensitized with 20 µg ovalbumin dissolved in PBS (OVA, #MFCD00889188, Macklin, China) and 1 mg Aluminium hydroxide (Al(OH)_3_ Macklin, China) in a total of 200 µL saline at day 1 and 8, then challenged with 5% OVA at day 15, 16 and 17 for 30 min. Mice in the control group were given the same dosage of PBS in saline.

### Animal treatments

The WT mice were randomly divided into three groups (*n* = 5): control group, OVA group and OVA + Ang-(1–7) group. The ATG5^−/−^ mice were randomly divided into two groups (*n* = 5): control group and OVA group. In the OVA + Ang-(1–7) group, mice were intranasally treated with 20 µL (30 µg/kg) of Ang-(1–7) [25] every day for 10 days prior to the challenge with OVA after sensitization at day 8. The treatment of mice was showed in Fig. 6A.

### Resistance to methacholine

After the last challenge with OVA for 24 h, the AHR of mice was measured according to previous reports [26]. The mice were anesthetized with sodium pentobarbital (40 mg/kg, intraperitoneal), and a tracheostomy tube was placed in the trachea of the mice. Airway resistance was measured at baseline and after the administration of nebulized methacholine (0, 12.5, 25, 50, and 100 mg/mL) using a Sci-Req FlexiVent machine (FX module 1, SCIREQ, Montreal, Canada).

### Cytokine levels in bronchioalveolar lavage fluids (BALFs)

After measurement of airway resistance, the BALFs were obtained through PBS (1 mL) injection via the tracheal tube. Supernatants were collected via centrifugation (× 800 g) for 15 min. Levels of IL-4 (#PI612, Beyotime, Shanghai, China), IL-5 (#PI620, Beyotime) and IL-13 (#PI539, Beyotime) in supernatants were measured using ELISA kits.

### Pathological observation

The pathological changes of mice were observed using hematoxylin and eosin (H&E) staining. According to previous reports [27], the lung tissues were fixed with 4% paraformaldehyde, dehydrated with gradient ethanol, embedded in paraffin and sectioned to slices (3 μm-thick). The slices were dewaxed with xylene and hydrated with gradient ethanol. After washing, the slices were stained with H&E solution (#G1120, Solarbio, China) for 5 min. After washing, the slices were dehydrated with gradient ethanol, cleared with xylene and sealed with neutral gum. Five fields of view were visualized at × 400 magnification using a light microscope (CKX53, Olympus, Japan). Area of inflammatory infiltration was used to assess the inflammation in airway. The area of inflammatory infiltration was analyzed using ImageJ software (v.2021, National Institutes of Health, Bethesda, MD, USA).

### Immunohistochemical staining

The slices were treated with H_2_O_2_, boiled twice, and washed according to reported descriptions [28]. The slices were incubated overnight with rabbit antibodies, α-SMA (1:800, #A17910, ABclonal), TGF-β1 (1:800, #A7040, ABclonal) and LC3B (1:800, (#MA5-37852, 1:800, ThermoFisher Scientific, China) at 4 °C. After washing, the slices were incubated with the secondary antibody (1:1000, AS029, ABclonal) for 120 min at 37 °C, then stained with diaminobenzidine, dehydrated, cleared and sealed. All analysis of immunohistochemical staining sections was performed using ImageJ software.

### Statistics

Data were analyzed using GraphPad Prism software (version 8.0, GraphPad Software Inc., La Jolla, CA, US). The results were shown as mean ± standard deviation. Data difference was evaluated using one-way analysis of variance following the Tukey’s post hoc test. Statistical significance was set at *p* < 0.05.

## Results

### Ang-(1–7) treatment suppressed ATG5 protein expression in IL-13-treated cells

**Fig. 1:**
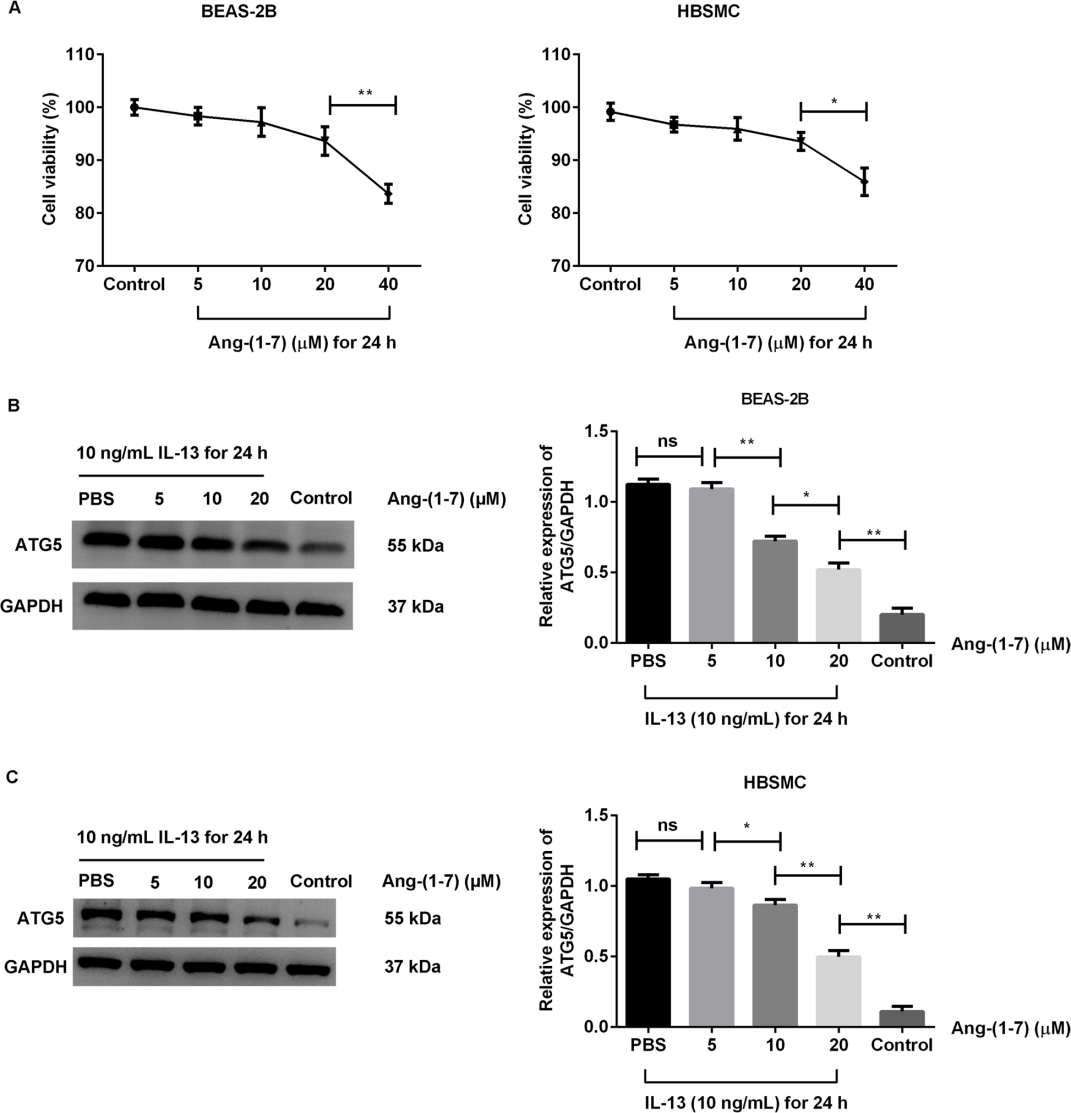
Effects of Ang-(1–7) on ATG5 protein expression in IL-13-treated BEAS-2B and HBSMC cells. **A** BEAS-2B and HBSMC cells were treated with different dose (5, 10, 20, 40 µM) of Ang-(1–7) for 24 h. Cell viability was measured via a CCK-8 kit. BEAS-2B and HBSMC cells were treated with 10 ng/mL human IL-13 for 24 h, then treated with different dose (5, 10, 20 µM) of Ang-(1–7) for 24 h. Expression of ATG5 protein in BEAS-2B cells (**B**) and HBSMC cells (**C**) was measured by western blotting. *p < 0.05, **p < 0.01. ns: no significance

As shown in Fig. 1A, the toxicity of Ang-(1–7) on BEAS-2B and HBSMC cells was observed. The cells were treated with different dose (5, 10, 20, 40 µM) of Ang-(1–7) for 24 h. The results showed that cell viability was significantly decreased after 40 µM Ang-(1–7) treatment for 24 h when compared with the control cells (*p* < 0.05). To avoid the effect of different cell viability on ATG5 expression, the 40 µM Ang-(1–7) was not chosen in next experiments. In the IL-13-treated cells, the ATG5 protein expression were significantly increased compared with the control cells (*p* < 0.01, Fig. 1B and C). Meanwhile, the level of ATG5 protein was decreased with increasing Ang-(1–7) doses (5, 10, 20 µM) in the IL-13-treated cells (Fig. 1B, C). Notably, the level of ATG5 protein was clearly decreased after 20 µM Ang-(1–7) treatment compared with the other dose (*p* < 0.01).

On the basis of cell viability and ATG5 protein, 20 µM Ang-(1–7) was the most effective concentration tested. The effects of 20 µM Ang-(1–7) treatment alone on secretion of cytokines were observed (Supplementary Fig. 3). The levels of IL-25 and IL-33 in BEAS-2B cells and the expression of TGF-β1 and α-SMA protein in HBSMC cells were observed. No significance was found among groups (*p* > 0.05).

### Effects of Ang-(1–7) treatment on IL-13-treated BEAS-2B cells

**Fig. 2:**
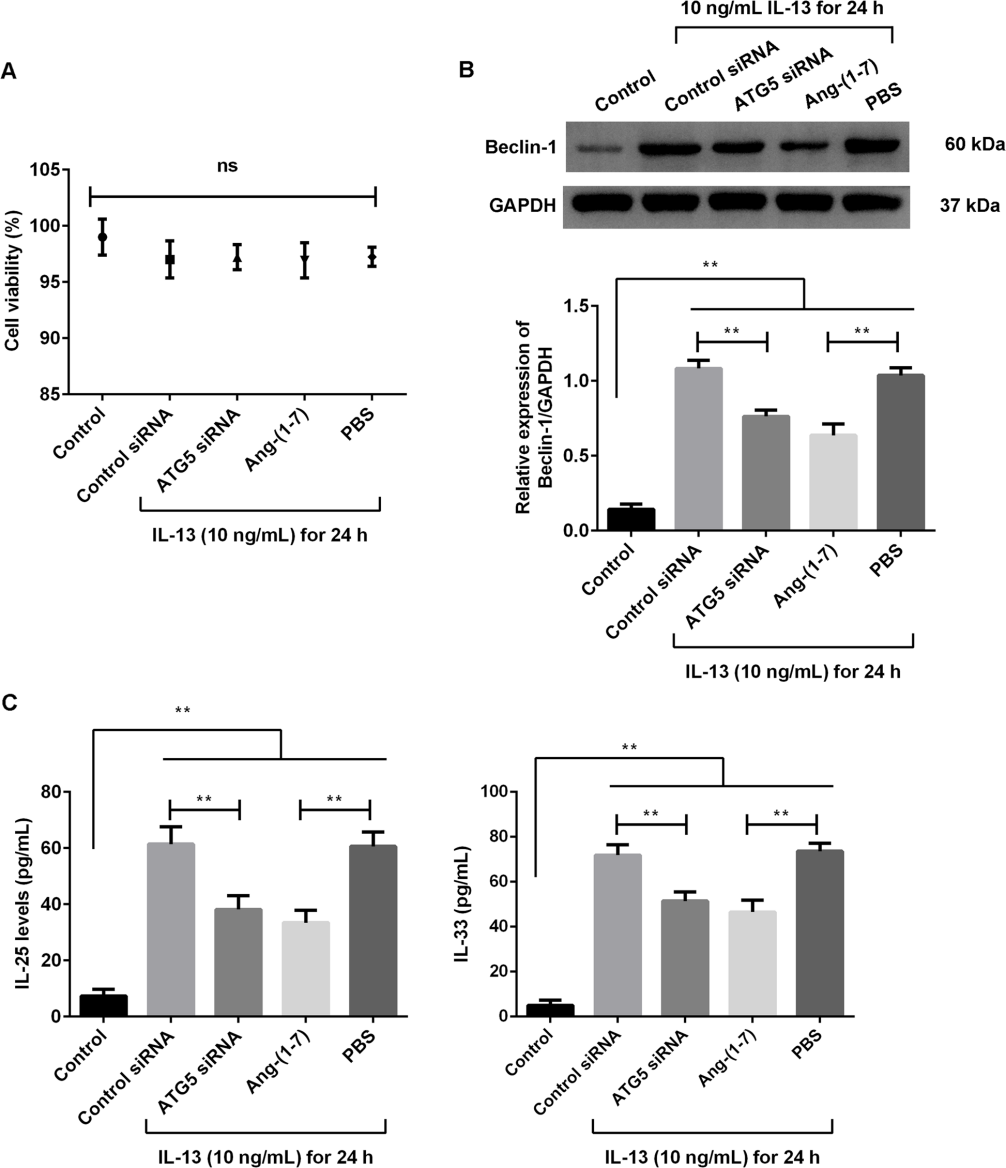
Ang-(1–7) treatment suppressed the expression of Beclin-1 protein and secretions of IL-25 and IL-33 in the IL-13-treated BEAS-2B cells. BEAS-2B cells were transfected with control siRNA or ATG5 siRNA, and then treated with IL-13 for 24 h. The IL-13-treated cells were treated with 20 µM Ang-(1–7) for 24 h, and the PBS treatment was as a control treatment. **A** Cell viability in different groups was measured using a CCK-8 kit. **B** Expression of Beclin-1 protein was measured using Western blotting. **C** Secretion of IL-25 and IL-33 was measured using ELISA kits. ns: no significance. *p < 0.05, **p < 0.01

The results of control siRNA or ATG5 siRNA transfection in the IL-13-treated cells were observed. Meanwhile, the results of 20 µM Ang-(1–7) treatment in the IL-13-treated cells were measured. The grouped data showed that there was no loss in cell viability following treatments (Fig. 2A, p > 0.05). For the Beclin-1 protein expression (Fig. 2B), the ATG5 siRNA or Ang-(1–7) treatment significantly suppressed its expression when contrasted to the control treatment in the IL-13-treated cells (*p* < 0.01). Meanwhile, the secretion of IL-25 and IL-33 (Fig. 2C) was significantly decreased after ATG5 siRNA or Ang-(1–7) treatment compared with the control treatment in the IL-13-treated cells (*p* < 0.01). These results showed that the effects of Ang-(1–7) treatment were similarly effective to ATG5 siRNA transfection at reducing autophagy-related protein expression as well as Th2 responses produced by BEAS-2B cells treated with IL-13.

### Effects of Ang-(1–7) treatment on IL-13-treated HBSMC cells

**Fig. 3:**
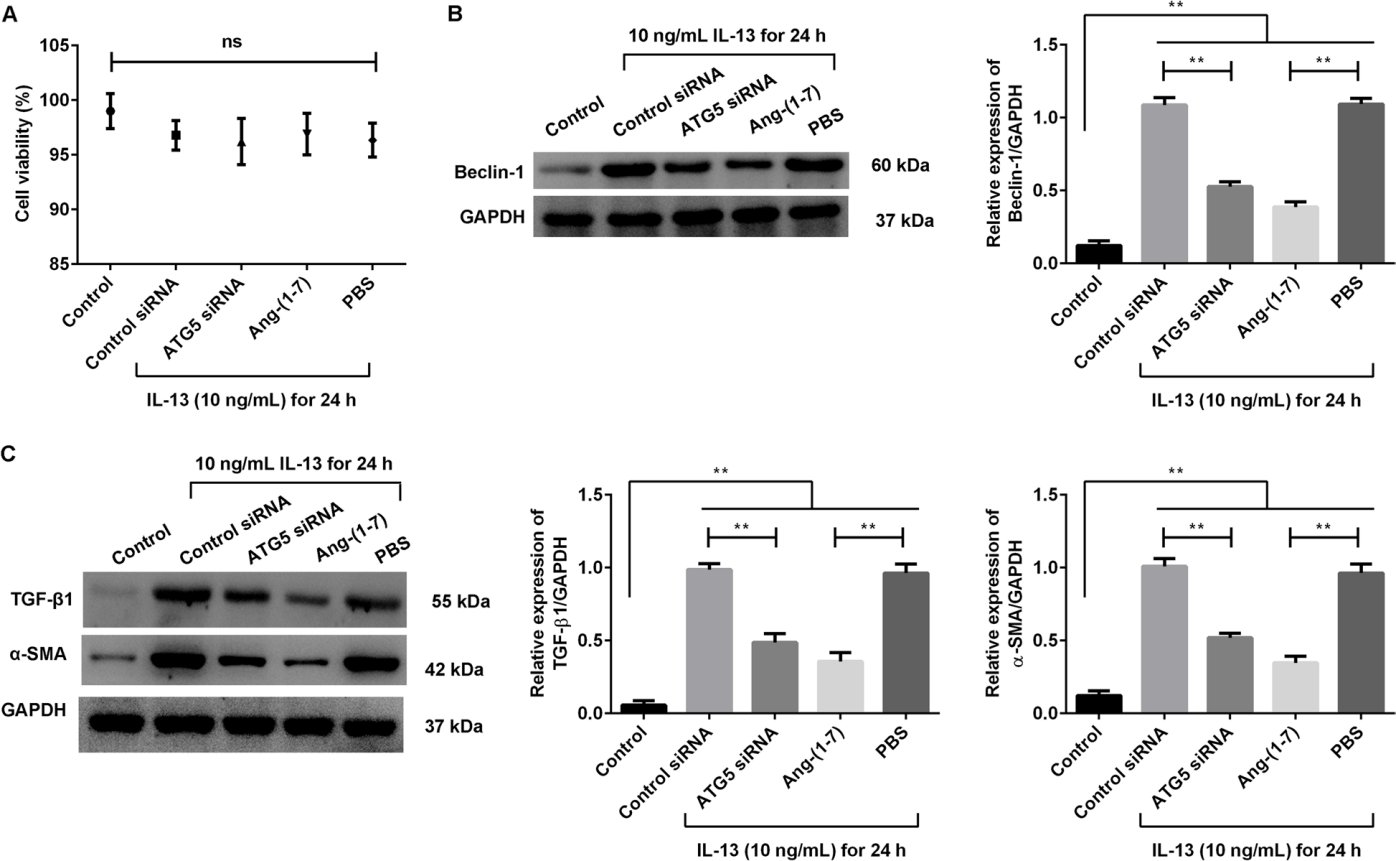
Ang-(1–7) treatment suppressed the expression of Beclin-1, TGF-β1 and α-SMA protein in the IL-13-treated HBSMC cells. HBSMC cells were transfected with control siRNA or ATG5 siRNA, and then treated with IL-13 for 24 h. The IL-13-treated cells were treated with 20 µM Ang-(1–7) for 24 h, and the PBS treatment was as a control treatment. **A** Cell viability was measured using a CCK-8 kit. **B** Expression of Beclin-1 protein was measured using Western blotting. **C** Expression of TGF-β1 and α-SMA protein was measured using Western blotting. ns: no significance. *p < 0.05, **p < 0.01

Similar to the treatment of BEAS-2B cells, the HBSMC cells were transfected with control siRNA or ATG5 siRNA and treated with IL-13 for 24 h. The effects of Ang-(1–7) were compared with the results of ATG5 siRNA transfection in the IL-13-treated cells. The grouped data showed that there was no loss in cell viability following treatments (Fig. 3A, p > 0.05). For the Beclin-1 protein expression (Fig. 3B), the ATG5 siRNA or Ang-(1–7) treatment significantly suppressed its expression when contrasted to the control treatment in the IL-13-treated cells (*p* < 0.01). Meanwhile, the expression of TGF-β1 and α-SMA protein (Fig. 3C) was significantly decreased after ATG5 siRNA or Ang-(1–7) treatment compared with the control treatment in the IL-13-treated cells (*p* < 0.01). These results showed that the effects of Ang-(1–7) treatment were similarly effective to ATG5 siRNA transfection at reducing autophagy-related protein expression as well as fibrosis-related protein expression produced by HBSMC cells treated with IL-13.

### Ang-(1–7) treatment suppressed the effects of ATG5-overexpressing in the IL-13-treated BEAS-2B and IL-13-treated HBMSC cells

**Fig. 4:**
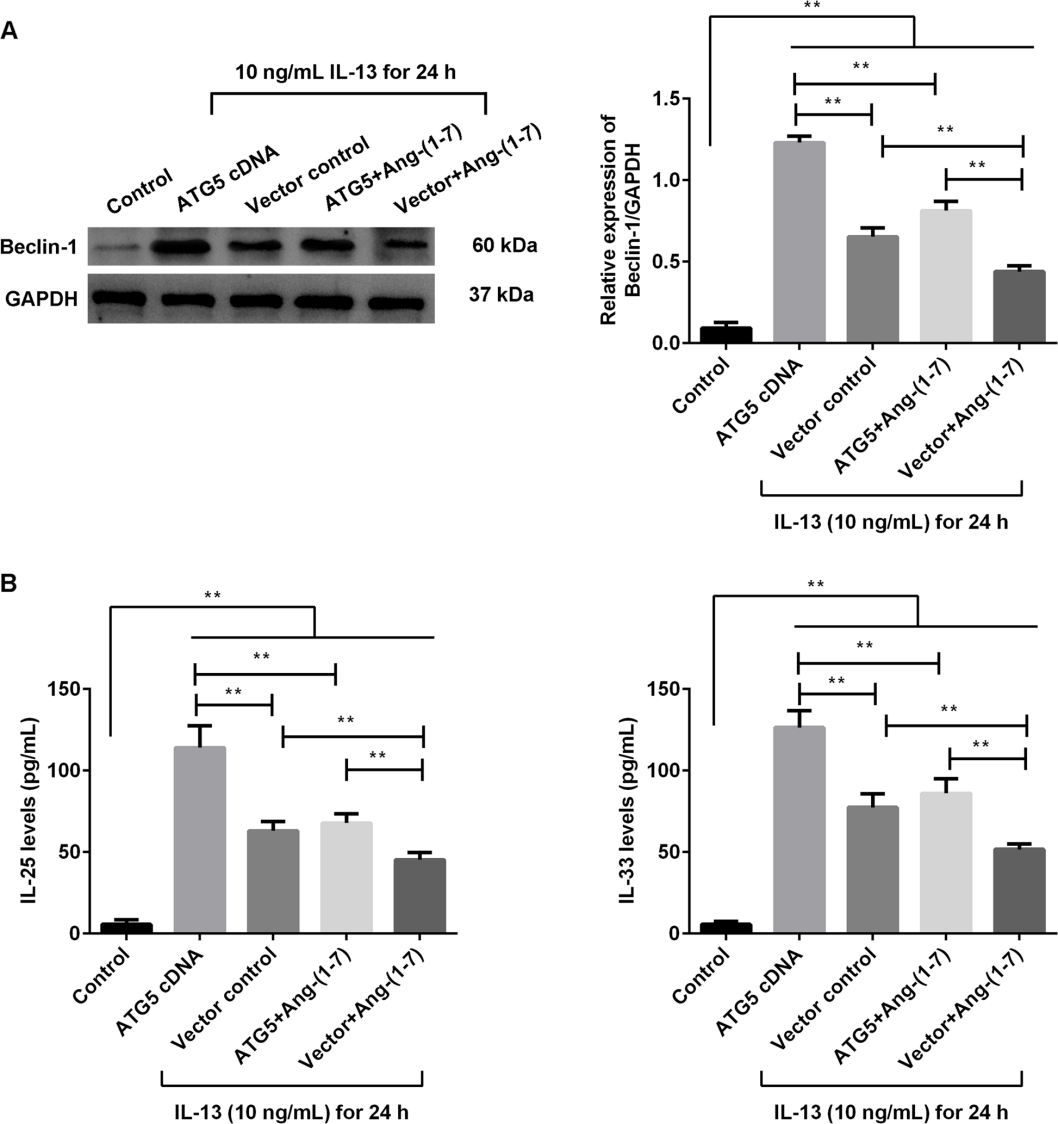
Ang-(1–7) suppressed the results of ATG5 overexpression in IL-13-induced BEAS-2B cells. The BEAS-2B cells were transfected with ATG5 cDNA or vector control, and treated with IL-13 for 24 h. The ATG5 cDNA-transfected or vector control-transfected cells were treated with 20 µM Ang-(1–7) treatment. **A** Expression of Beclin-1 protein was measured using Western blotting. **B** Secretion of IL-25 and IL-33 was measured using ELISA kits. **p < 0.01

**Fig. 5:**
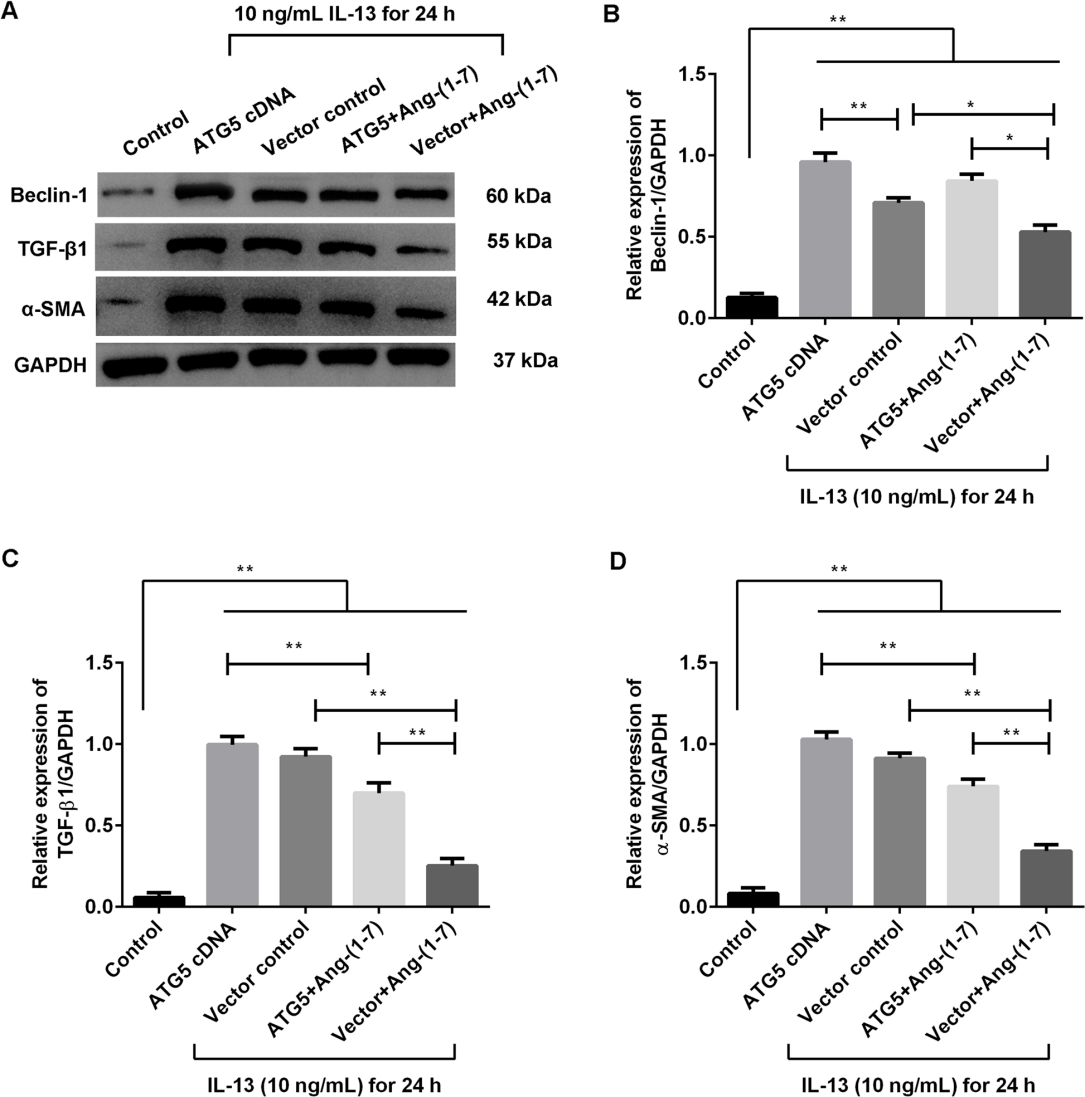
Ang-(1–7) suppressed the results of ATG5 overexpression in IL-13-induced HBSMC cells. The HBSMC cells were transfected with ATG5 cDNA or vector control, and treated with IL-13 for 24 h. The ATG5 cDNA-transfected or vector control-treated cells were treated with 20 µM Ang-(1–7) treatment. **A** Expression of Beclin-1, TGF-β1 and α-SMA protein was measured using Western blotting. **B** Relative expression of Beclin-1 protein was normalized by GAPDH. **C** Relative expression of TGF-β1 protein was normalized by GAPDH. **D** Relative expression of α-SMA protein was normalized by GAPDH. *p < 0.05, **p < 0.01

The cells were transfected with ATG5 cDNA or vector control, and treated with IL-13 for 24 h. Meanwhile, the effects of Ang-(1–7) treatment on the transfected cells were observed. In the BEAS-2B cells (Fig. 4), the grouped data showed that the expression of Beclin-1 protein was significantly increased after ATG5 cDNA transfection compared with the control transfection in the IL-13-treated cells (Fig. 4A, p < 0.01). The secretion of IL-25 and IL-33 was significantly increased after ATG5 cDNA transfection compared with the control transfection in the IL-13-treated cells (Fig. 4B, p < 0.01). However, the effects of ATG5 cDNA transfection were clearly suppressed by the Ang-(1–7) administration (*p* < 0.01). In the HBSMC cells (Fig. 5), the expression of Beclin-1, TGF-β1 and α-SMA protein was significantly increased after ATG5 cDNA transfection compared with the control transfection in the IL-13-treated cells (Fig. 5A-D, p < 0.01). The Ang-(1–7) administration also significantly hampered the effects of ATG5 cDNA transfection in the IL-13-treated HBSMC cells (*p* < 0.01). These results showed that Ang-(1–7) treatment suppressed the results of ATG5 overexpressing in the IL-13-induced cells.

### Ang-(1–7) treatment or ATG5 knockout suppressed airway inflammation in OVA-induced mice

**Fig. 6:**
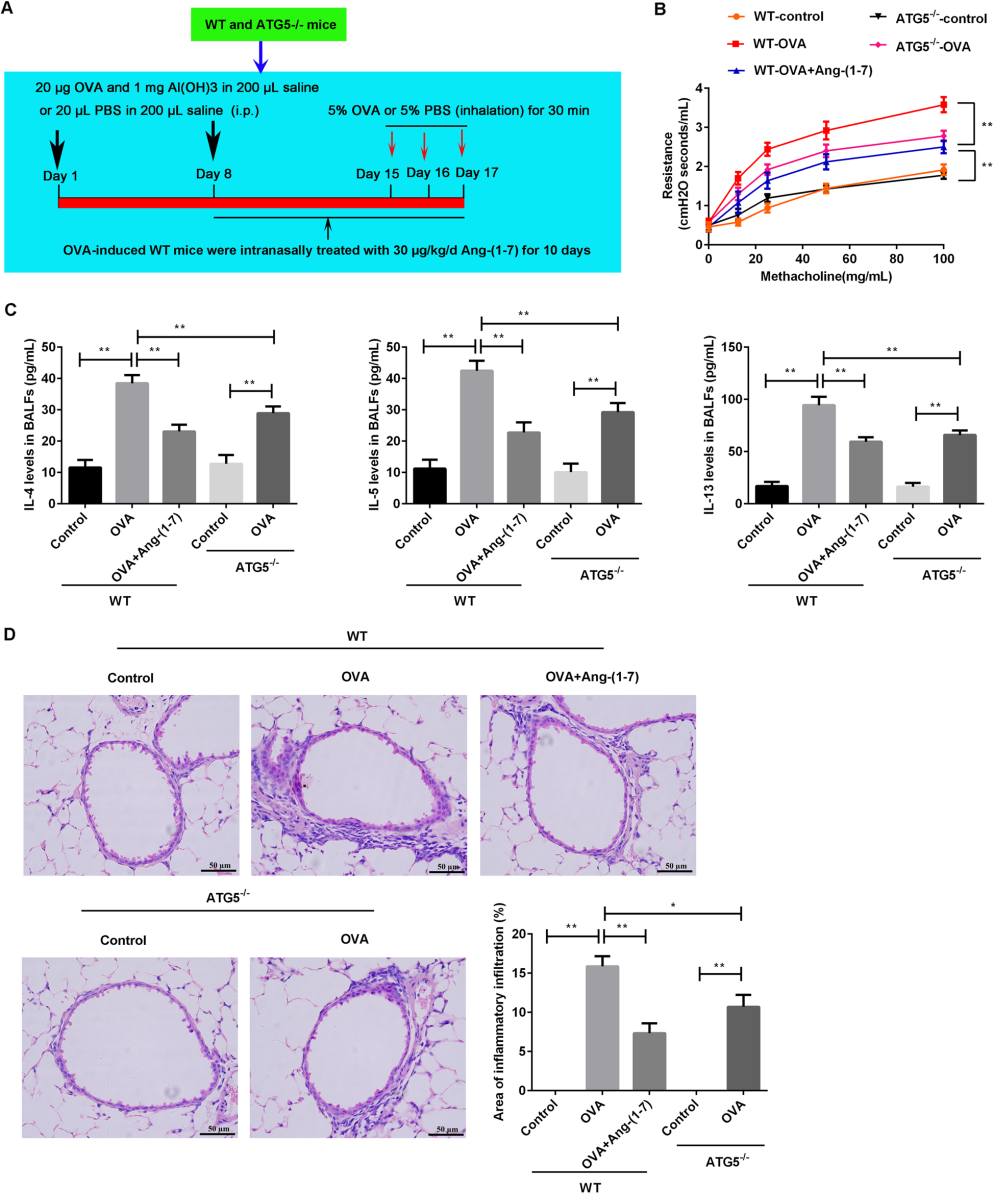
Ang-(1–7) treatment or ATG5 knockout suppressed airway inflammation in OVA-challenged mice. **A** The diagram of animal treatments. An allergic asthma model was induced by OVA in WT mice and ATG5.^−/−^ mice. The OVA-induced WT mice were treated with 30 µg/kg/d Ang-(1–7) for 10 days after sensitization at day 8. **B** Airway resistance to methacholine were measured. **C** Levels of IL-4, IL-5 and IL-33 in BALFs were measured using ELISA kits. **D** Observation of pathological changes by H&E staining. Area of inflammatory infiltration in airway was analyzed using ImageJ software. **p < 0.01

To further analyze the effects of Ang-(1–7) on ATG5-mediated autophagy in allergic asthma, the OVA-challenged WT mice were treated with Ang-(1–7) and the OVA-challenged ATG5^−/−^ mice were as a control (Fig. 6A). In the two genotypes, the airway resistance (Fig. 6B), Th2 inflammatory factors (IL-4, IL-5 and IL-33) in BALFs (Fig. 6C) and area of inflammatory infiltration in airway (Fig. 6D) were significantly upregulated in the OVA-treated mice compared with the control mice (*p* < 0.01). Compared with the OVA-treated WT mice, Ang-(1–7) treatment or ATG5 knockout markedly decreased the airway resistance, levels of Th2 inflammatory factors and area of inflammatory infiltration (*p* < 0.01). These results showed that the effects of Ang-(1–7) treatment on airway inflammation were similar to ATG5 knockout in the OVA-induced mice.

### Ang-(1–7) treatment or ATG5 knockout suppressed airway remodeling in OVA-induced mice

**Fig. 7:**
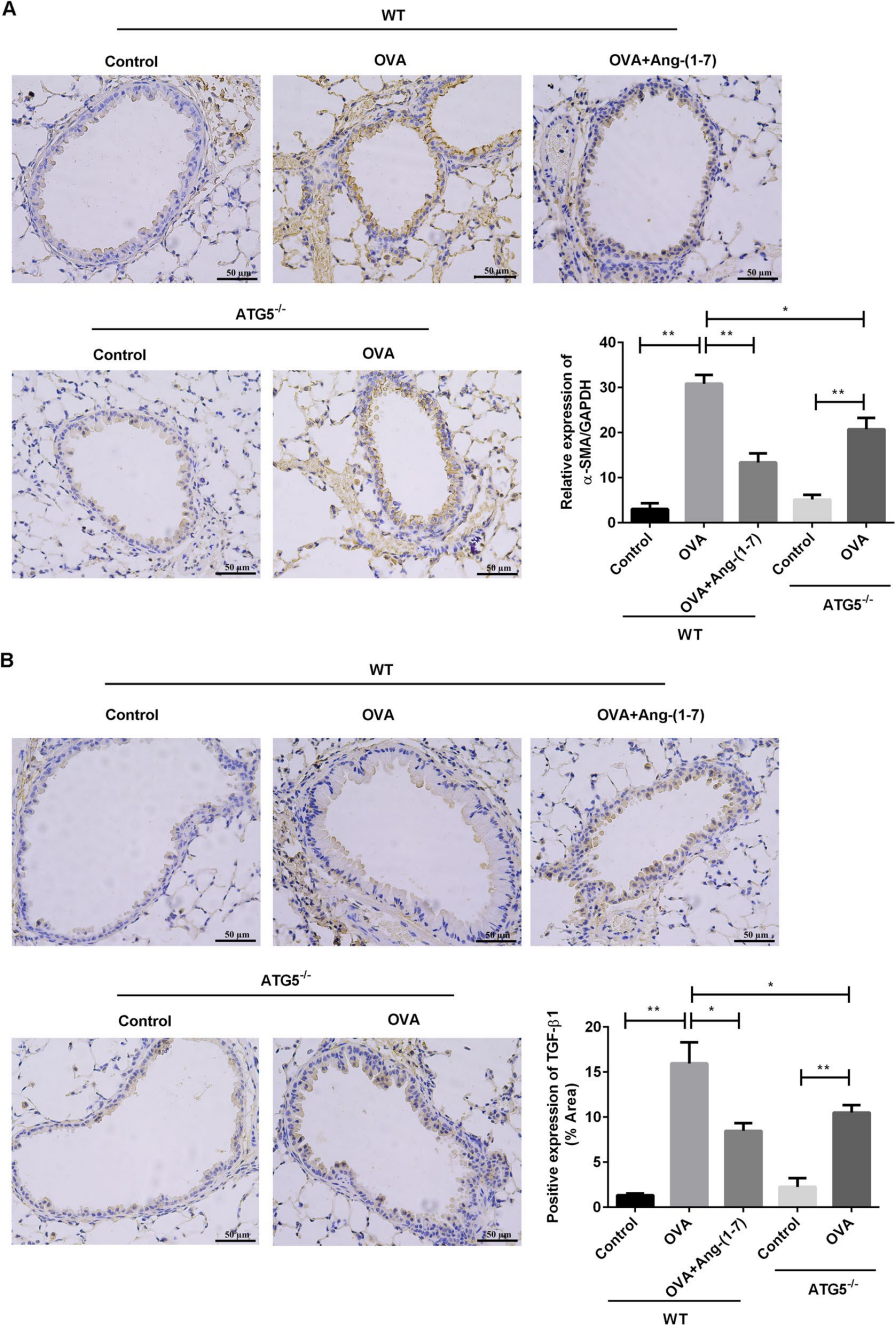
Ang-(1–7) treatment or ATG5 knockout suppressed airway remodeling in by OVA-challenged mice. Expression of α-SMA (**A**) and TGF-β1 (**B**) in airway was analyzed by immunohistochemical analysis. Expression of results was analyzed by ImageJ software. *p < 0.05, **p < 0.01

Furthermore, the airway remodeling was assessed through observing the expression of α-SMA and TGF-β1 in the WT mice and ATG5^−/−^ mice (Fig. 7). In the two genotypes, the expression of α-SMA (Fig. 7A) and TGF-β1 (Fig. 7B) was significantly increased after OVA treatment compared with the control mice (*p* < 0.01). Remarkably, the expression of α-SMA and TGF-β1 was lower in the OVA-induced ATG5^−/−^ mice compared with that in the OVA-induced WT mice (*p* < 0.05). In the OVA-treated WT mice, Ang-(1–7) treatment significantly declined the expression of α-SMA and TGF-β1 (*p* < 0.05). These results showed that the effects of Ang-(1–7) treatment on airway remodeling were similar to ATG5 knockout in the OVA-induced mice.

### Ang-(1–7) treatment or ATG5 knockout suppressed airway autophagy in OVA-induced mice

**Fig. 8:**
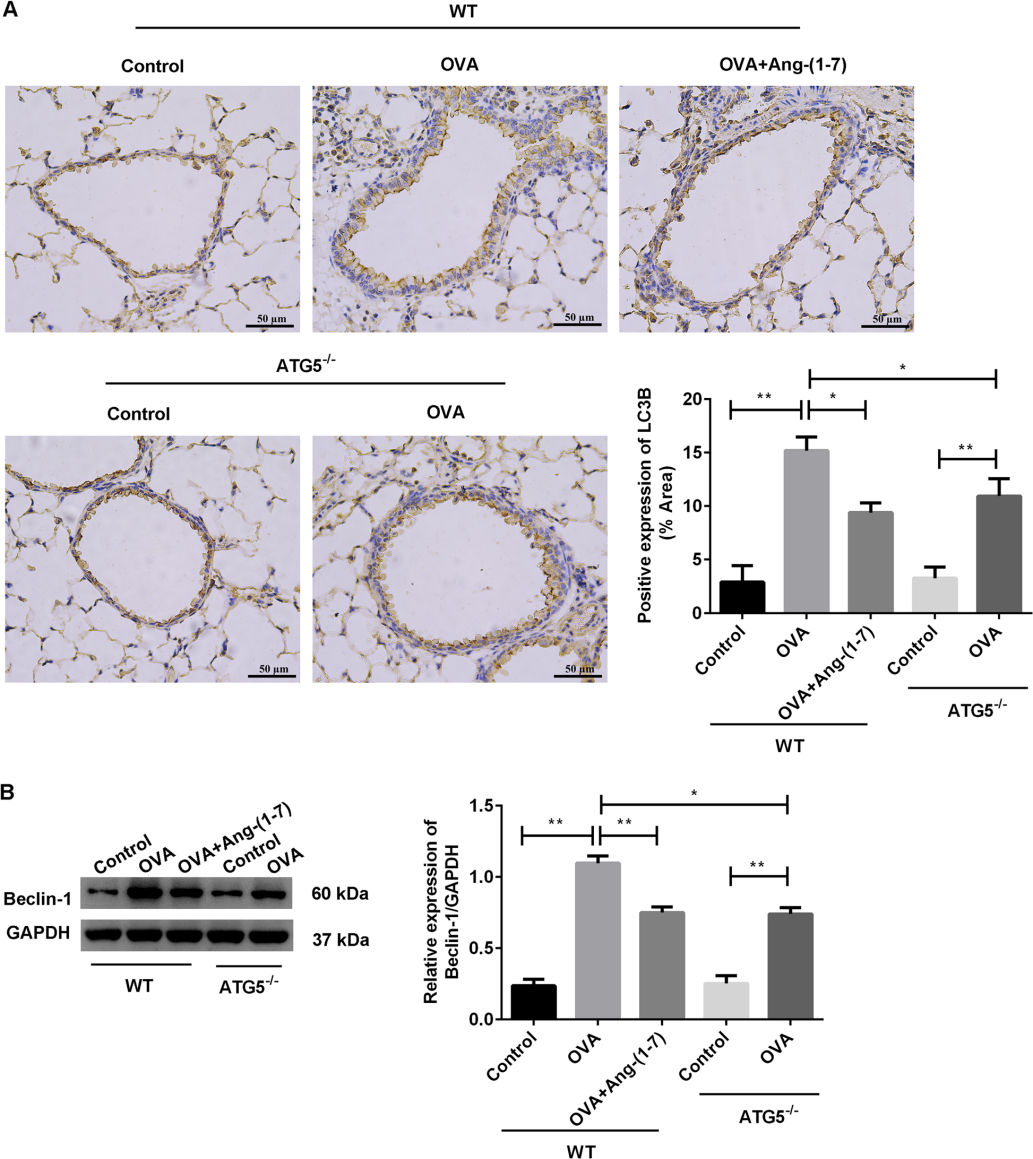
Ang-(1–7) treatment or ATG5 knockout suppressed airway autophagy in OVA-challenged mice. **A** Expression of LC3B in airway was evaluated by immunohistochemical analysis. **B** Expression of Beclin-1 in airway was evaluated by western blotting. *p < 0.05, **p < 0.01

The airway autophagy-related proteins LC3B and Beclin-1 were evaluated (Fig. 8) in the WT mice and ATG5^−/−^ mice. In the two genotypes, the expression of LC3B (Fig. 8A) and Beclin-1 (Fig. 8B) was obviously increased after OVA treatment compared with the control mice (*p* < 0.01). Remarkably, the expression of LC3B and Beclin-1 was lower in the OVA-induced ATG5^−/−^ mice compared with that in the OVA-induced WT mice (*p* < 0.05). In the OVA-induced WT mice, Ang-(1–7) treatment significantly declined the expression of LC3B and Beclin-1 (*p* < 0.01). These results showed that the effects of Ang-(1–7) treatment on airway autophagy were similar to ATG5 knockout in the OVA-induced mice.

## Discussion

Here, Ang-(1–7) treatment suppressed the secretion of IL-25 and IL-33 in the IL-13-treated BEAS-2B cells, and inhibited the expression of TGF-β1 and α-SMA protein in the IL-13-treated HBSMC cells. Meanwhile, Ang-(1–7) treatment suppressed the expression of ATG5 and Beclin-1 protein in the IL-13-treated BEAS-2B cells and IL-13-treated HBSMC cells. Importantly, the effects of Ang-(1–7) were similar to the ATG5 suppression in the IL-13-treated cells. Ang-(1–7) treatment suppressed the effects of ATG5 overexpression in the IL-13-treated cells. These results showed that Ang-(1–7) treatment played its roles via suppressing ATG5 in the IL-13-treated cells. In allergic asthma, IL-13 is a critical cytokine in the induction of the pathogenic Th2 response [29]. IL-13 is involved in the regulation of IgE synthesis, mucus hypersecretion, subepithelial fibrosis and eosinophil infiltration. It has shown that IL-13 induces features of the allergic response via its actions on epithelial and smooth muscle cells [30]. Therefore, 10 ng/mL human IL-13 was used to induce cell model in this study.

Ang-(1–7) has been reported to improve allergic asthma by suppressing eosinophils, Th2 cytokine secretion and macrophage infiltration, and downregulating the phosphorylation of CCL2, mitogen-activated protein kinase (MAPK) and nuclear factor kappa-B (NF-κB) pathways [14–17]. TGF-β1, α-SMA and collagens are fibrotic-related cytokines which promote airway remodeling in asthma [14, 16, 31]. Ang-(1–7) rescued chronic intermittent hypoxia-aggravated TGF-β-mediated epithelial to mesenchymal transition to suppress expression of α-SMA and collagen IV in airway epithelia [16]. Our findings were in keeping with these earlier findings, that Ang-(1–7) treatment suppressed the secretion of Th2 cytokines (IL-4, IL-5 and IL-13) and the expression of TGF-β1 and α-SMA in the OVA-induced mice. Furthermore, Ang-(1–7) treatment suppressed the expression of ATG5-mediated autophagy protein (LC3-B and Beclin-1) in the OVA-induced mice.

As one of autophagy proteins, ATG5 knockout can lead to a decrease of Th2 cytokines secretion by type 2 innate lymphoid cells (ILC2s) in IL-33-induced mice that further inhibits NF-κB pathway transduction [32]. The ILC2s can be activated by exogenous signals, such as IL-33 and IL-25, and can secrete the Th2 cytokines, such as IL-5, IL-4 and IL-13 [33–35]. LC3-B is a marker of ATG5-dependent autophagy, and Beclin-1 has been shown to play a vital role in ATG5-dependent and ATG5-independent autophagy [36]. Autophagy is dysregulated in asthma, and ATG5 has attracted wide attentions as a representative gene of autophagy [13, 37]. In OVA-induced ATG5^−/−^ mice, the imbalance of CD4^+^ T lymphocyte subsets (Th1/Th2 and Treg/Th17) was improved, and the inflammation and airway remodeling were decreased [13]. Here, Ang-(1–7) inhalation was similarly effective to ATG5 knockout at reducing the airway inflammation, remodeling and autophagy in OVA-induced mice. These results suggest that suppression of ATG5-mediated autophagy is one mechanism of Ang-(1–7) treatment on reducing airway inflammation and airway remodeling in OVA-challenged mice.

Ang-(1–7) is an endogen peptide of renin-angiotensin system (RAS) acting through the Mas receptor [16, 38]. Cumulating evidences point to RAS has implicated in the regulation of inflammation and fibrosis in pulmonary diseases [38]. Targeting Ang-(1–7)/Mas receptor pathway may be a potential therapy for alleviating allergic airway inflammation, AHR and airway remodeling. However, the precise pharmacological mechanism of Ang-(1–7) and its potential toxicity in human requires further investigation. For higher efficacy, lower doses requirement and reduced systemic side effects, it will be more challenging requiring innovative technology to deliver Ang-(1–7) directly into the lung. The barrier between preclinical and clinical studies is often in that animal models lack the robustness to capture the complexity and relevance to human diseases [38]. It is hard to find a panacea with just a single-target approach. A combination of existing drugs and Ang-(1–7) is further required to observe the therapeutic effects for allergic asthma.

## Conclusions

In this study, we found that Ang-(1–7) suppressed airway inflammation and remodeling in allergic asthma through inhibiting ATG5-mediated autophagy. Our results contribute to explain an underlying mechanism of Ang-(1–7) for allergic asthma treatment.

### Supplementary Information

**Additional file 1: Supplementary figure 1.** The results of control siRNA or ATG5 siRNA transfection were confirmed by western blotting. (a) Expression of ATG5 protein in BEAS-2B cells. (b) Expression of ATG5 protein in HBSMC cells. ns: no significance, **p<0.01. **Supplementary figure 2.** The results of vector control or ATG5 cDNA transfection were confirmed by western blotting. (a) Expression of ATG5 protein in BEAS-2B cells. (b) Expression of ATG5 protein in HBSMC cells. ns: no significance, **p<0.01. **Supplementary figure 3.** Effects of 20 µM Ang-(1-7) treatment alone on secretion of cytokines in cells. (a) Levels of IL-25 and IL-33 in BEAS-2B cells. (b) Expression of TGF-β1 and α-SMA protein in HBSMC cells. ns: no significance.

**Additional file 2: **Full length of blots in figures.

## Data Availability

All data generated or analyzed during this study are included in this article. Further enquiries can be directed to the corresponding author.
