# Supplementary material for: Angiotensin-(1–7) suppresses airway inflammation and airway remodeling via inhibiting ATG5 in allergic asthma
Source: BMC Pulm Med. 2023 Nov 2;23:422. doi: 10.1186/s12890-023-02719-7 (PMC10623740; doi:10.1186/s12890-023-02719-7)

Supplementary figure 1: The results of control siRNA or ATG5 siRNA transfection were confirmed by western blotting. (a) Expression of ATG5 protein in BEAS-2B cells. (b) Expression of ATG5 protein in HBSMC cells. ns: no significance, **p<0.01.

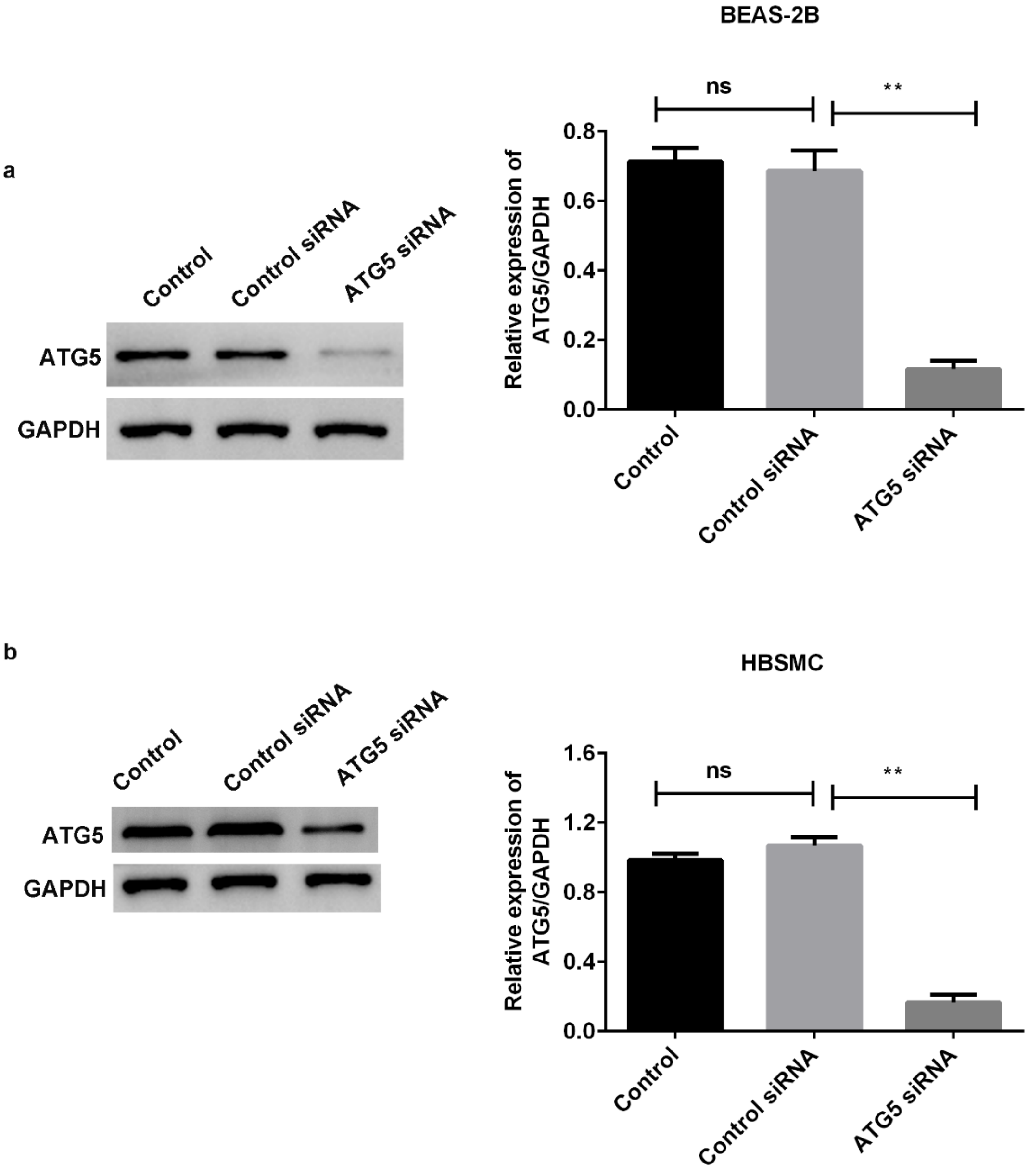

Supplementary figure 2: The results of vector control or ATG5 cDNA transfection were confirmed by western blotting. (a) Expression of ATG5 protein in BEAS-2B cells. (b) Expression of ATG5 protein in HBSMC cells. ns: no significance, **p<0.01.

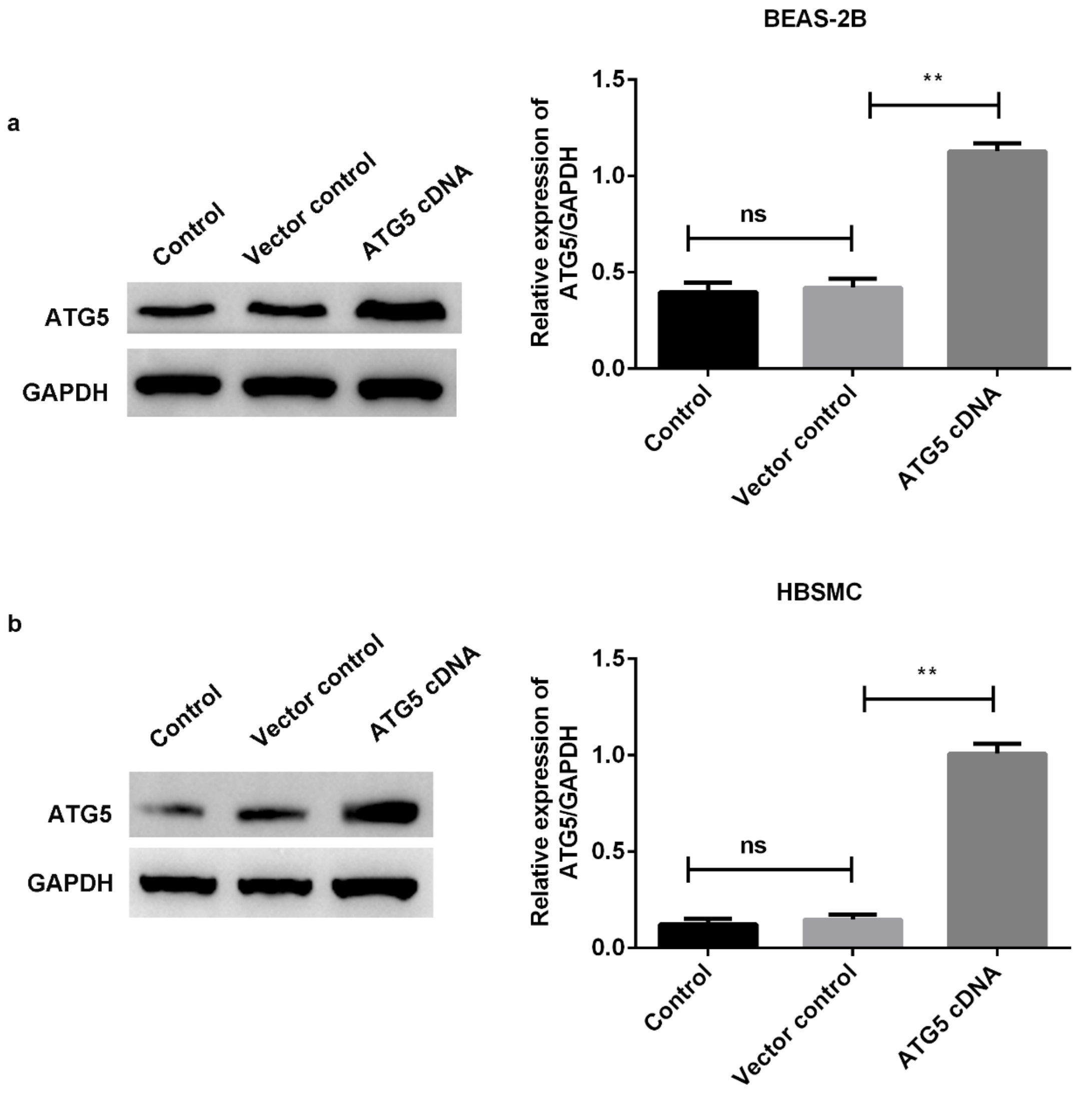

Supplementary figure 3: Effects of 20 μM Ang-(1-7) treatment alone on secretion of cytokines in cells. (a) Levels of IL-25 and IL-33 in BEAS-2B cells. (b) Expression of TGF-β1 and α-SMA protein in HBSMC cells. ns: no significance.

a

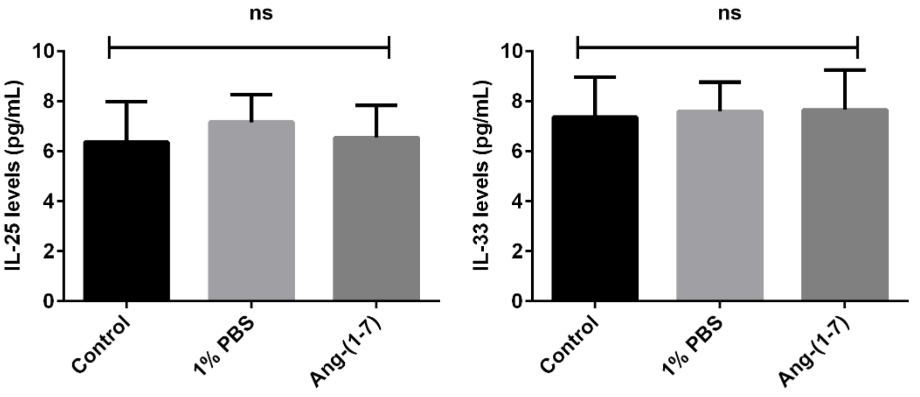

b

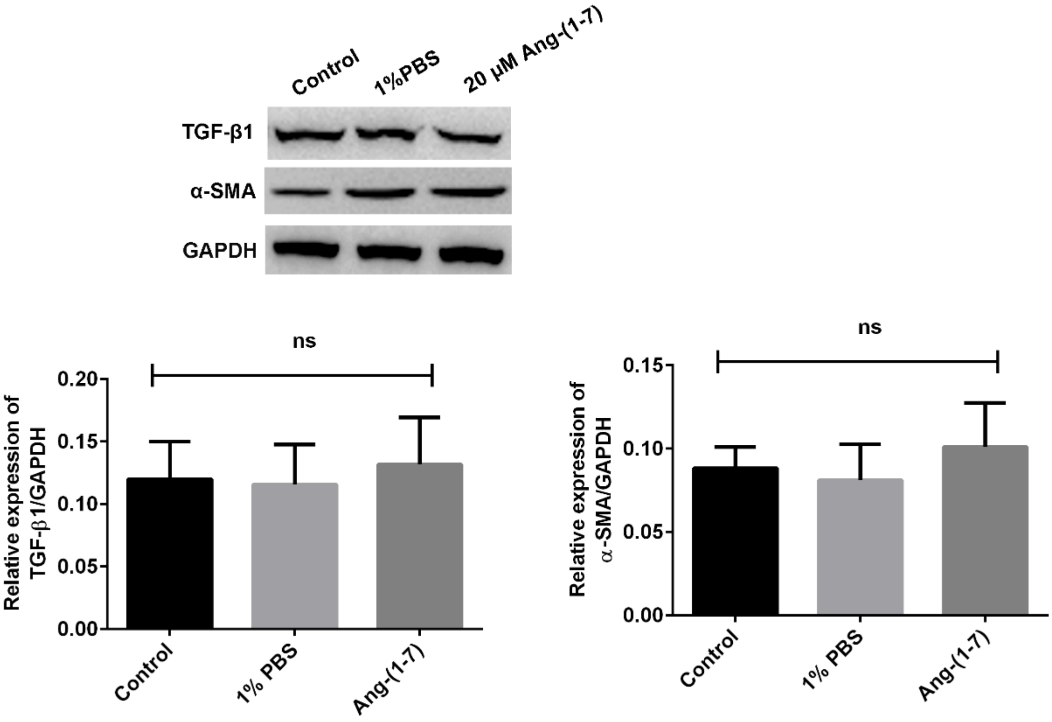

Supplement: Supplementary file 1 — Additional file 1: Supplementary figure 1. The results of control siRNA or ATG5 siRNA transfection were confirmed by western blotting. (a) Expression of ATG5 protein in BEAS-2B cells. (b) Expression of ATG5 protein in HBSMC cells. ns: no significance, **p<0.01. Supplementary figure 2. The results of vector control or ATG5 cDNA transfection were confirmed by western blotting. (a) Expression of ATG5 protein in BEAS-2B cells. (b) Expression of ATG5 protein in HBSMC cells. ns: no significance, **p<0.01. Supplementary figure 3. Effects of 20 µM Ang-(1-7) treatment alone on secretion of cytokines in cells. (a) Levels of IL-25 and IL-33 in BEAS-2B cells. (b) Expression of TGF-β1 and α-SMA protein in HBSMC cells. ns: no significance. [file 12890_2023_2719_MOESM1_ESM.pdf]
