# Supplementary material for: Angiotensin-(1–7) suppresses airway inflammation and airway remodeling via inhibiting ATG5 in allergic asthma
Source: BMC Pulm Med. 2023 Nov 2;23:422. doi: 10.1186/s12890-023-02719-7 (PMC10623740; doi:10.1186/s12890-023-02719-7)
Supplement: Supplementary file 2 — Additional file 2: Full length of blots in figures. [file 12890_2023_2719_MOESM2_ESM.pdf]

Figure 1B

ATG5

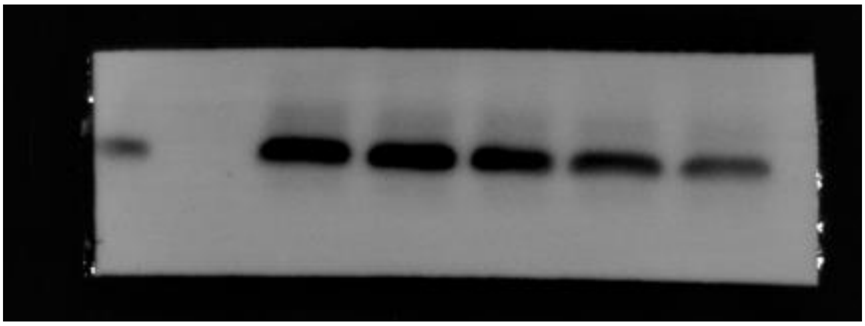

55 kDa

GAPDH

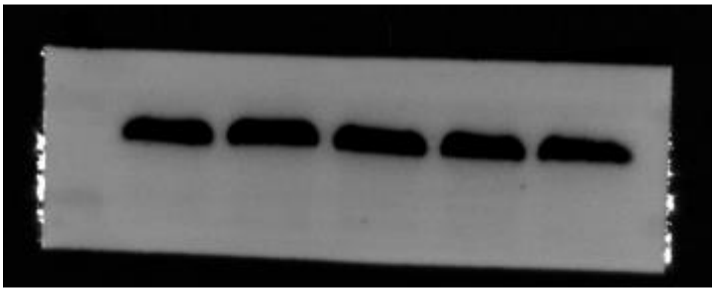

37 kDa

Figure 1C

ATG5

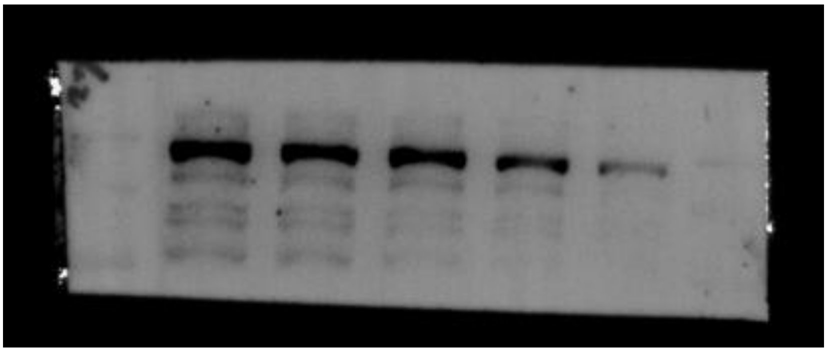

55 kDa

GAPDH

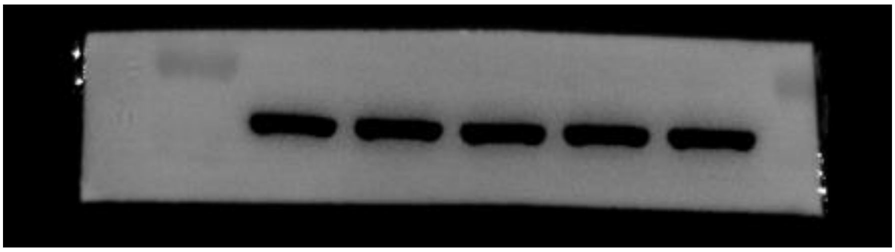

37 kDa

Figure 2B

Beclin-1

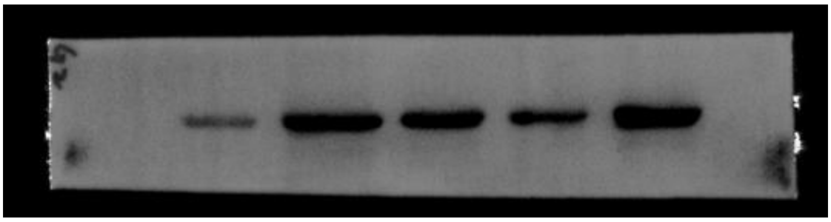

60 kDa

GAPDH

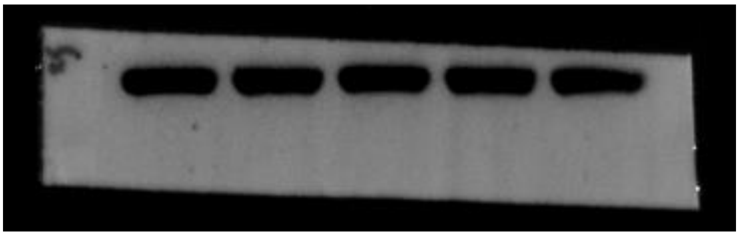

37 kDa

Figure 3B

Beclin-1

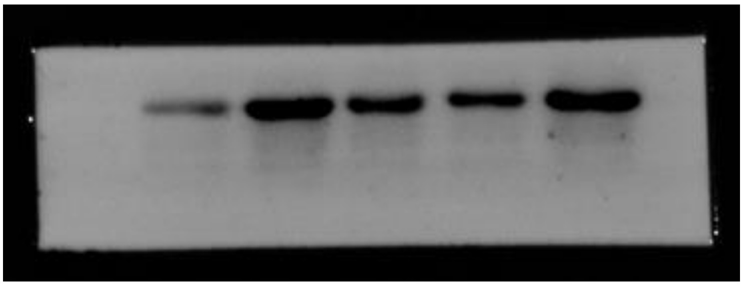

60 kDa

GAPDH

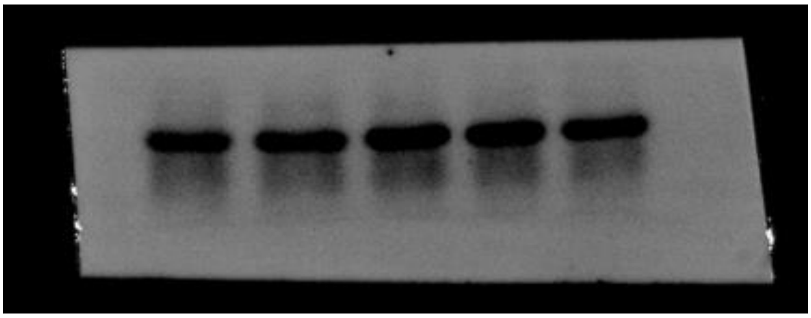

37 kDa

Figure 3C

TGF-β1

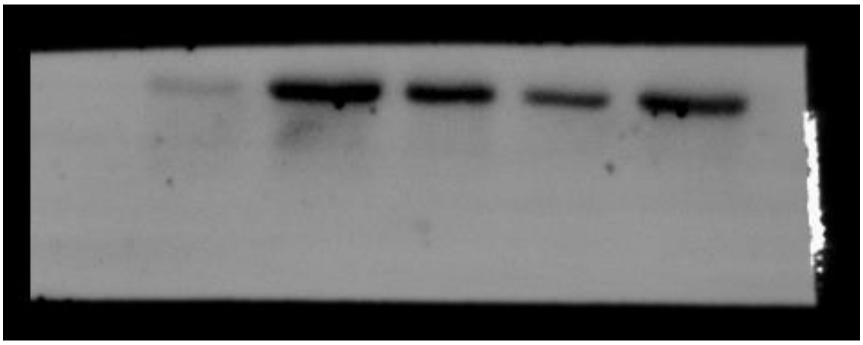

55 kDa

α-SMA

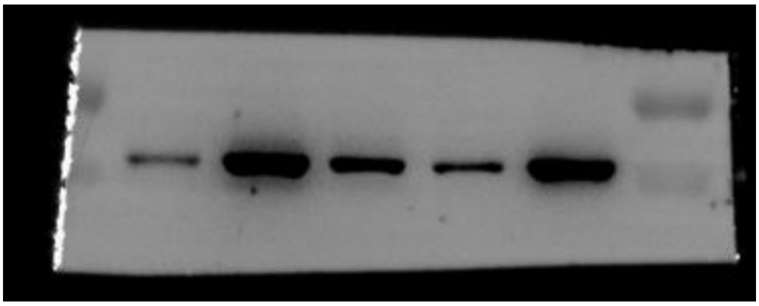

42 kDa

GAPDH

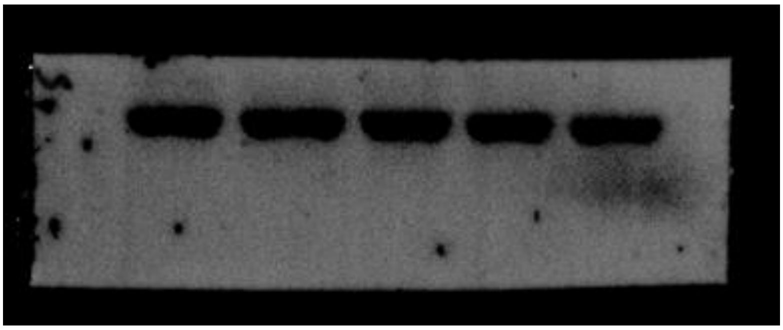

37 kDa

Figure 4A

Beclin-1

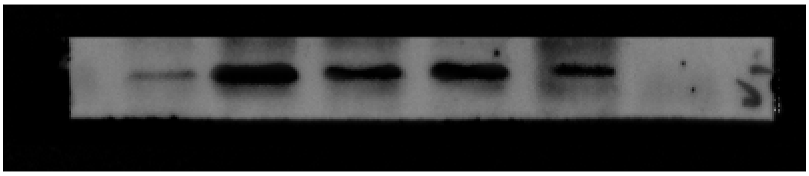

60 kDa

GAPDH

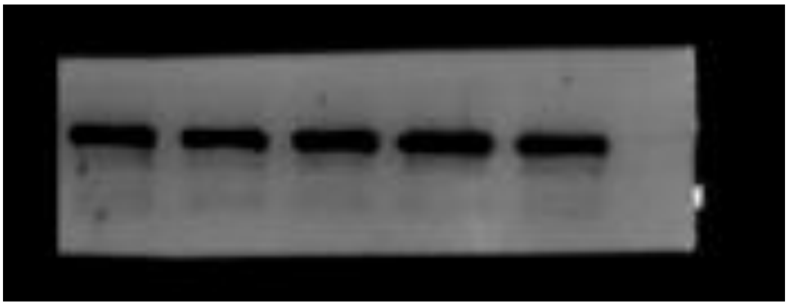

37 kDa

Figure 5A

Beclin-1

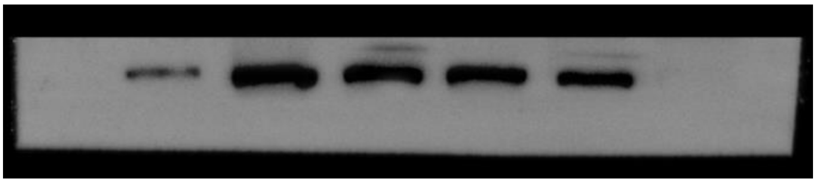

60 kDa

TGF-β1

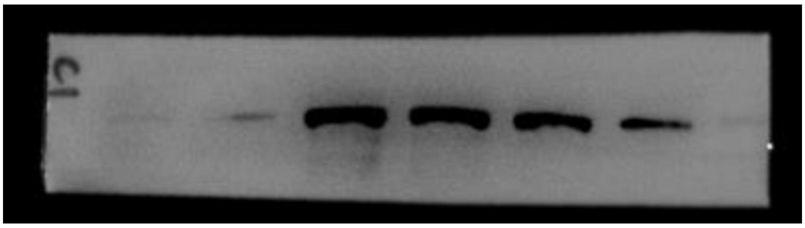

55 kDa

α-SMA

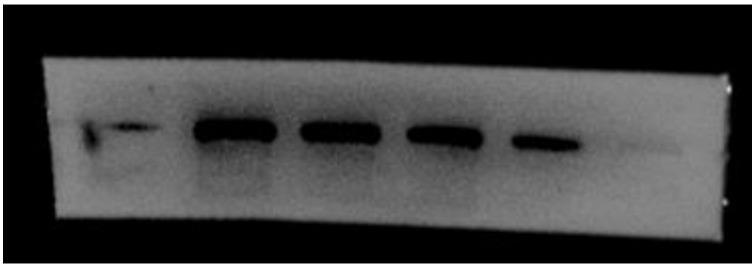

42 kDa

GAPDH

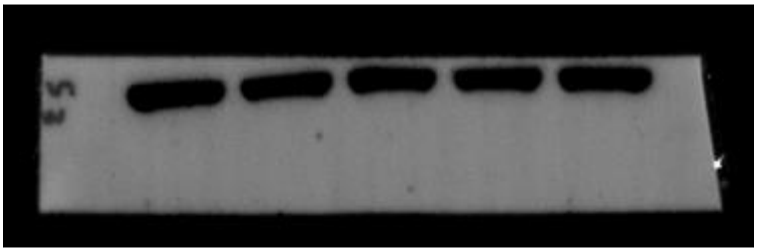

37 kDa

Figure 8B

Beclin-1

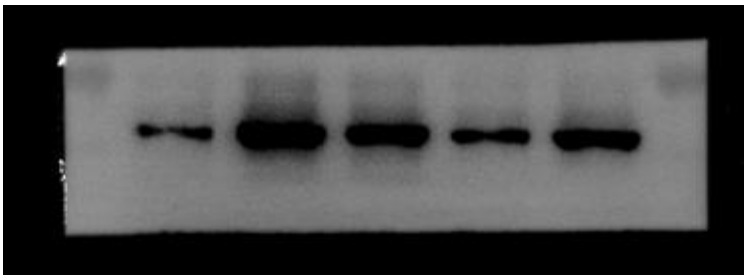

60 kDa

GAPDH

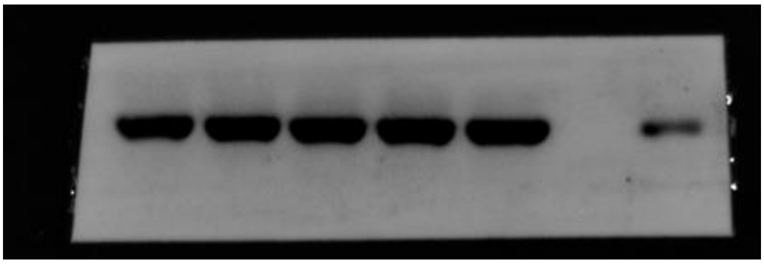

37 kDa

Supplementary figure 1a

ATG5

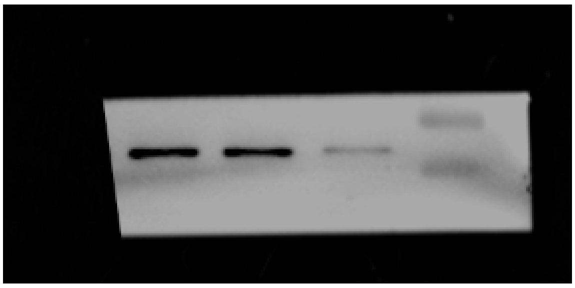

55 kDa

GAPDH

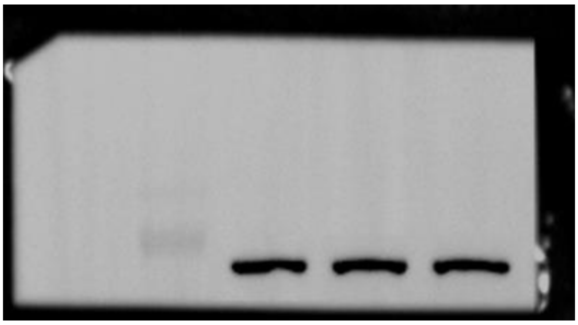

37 kDa

Supplementary figure 1b

ATG5

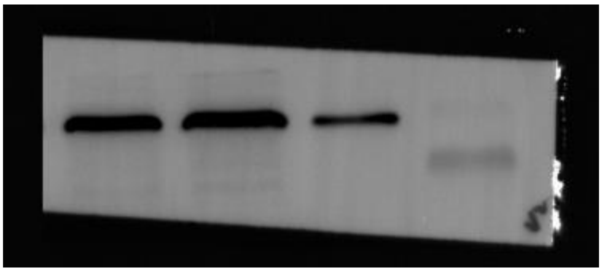

55 kDa

GAPDH

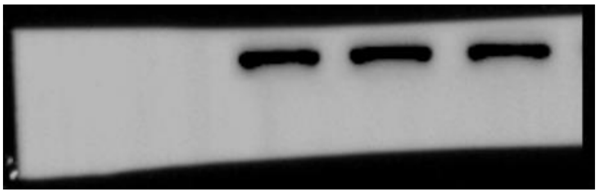

37 kDa

Supplementary figure 2 a

ATG5

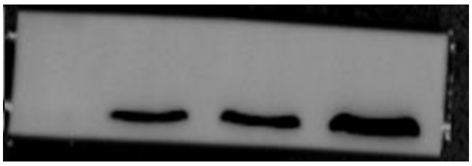

55 kDa

GAPDH

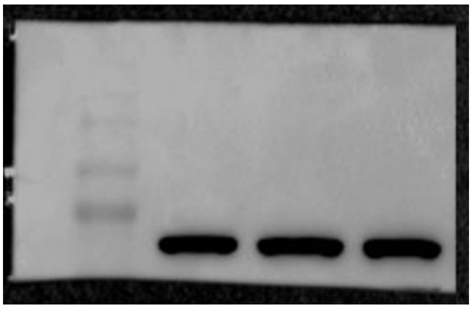

37 kDa

Supplementary figure 2 b

ATG5

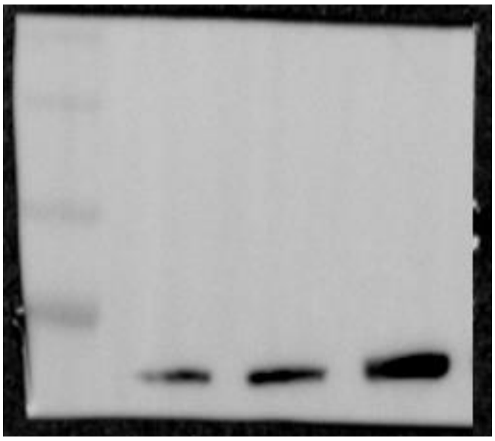

55 kDa

GAPDH

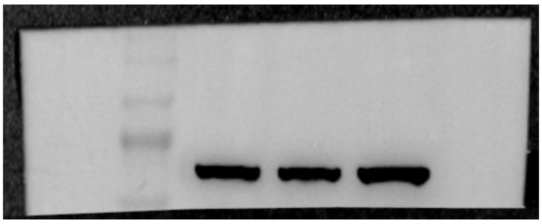

37 kDa

Supplementary figure 3b

TGF-β1

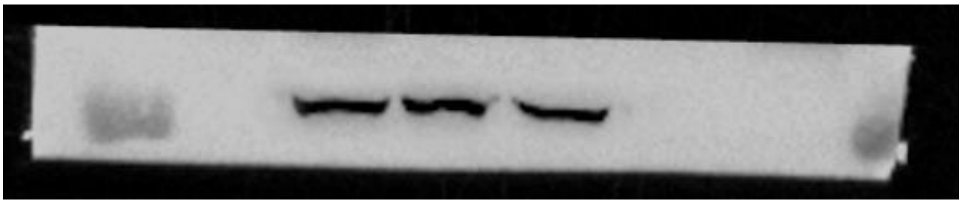

55 kDa

α-SMA

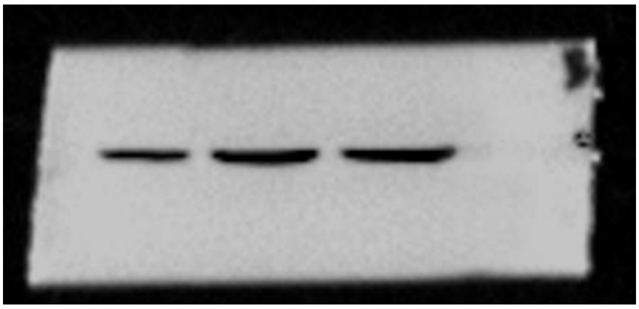

42 kDa

GAPDH

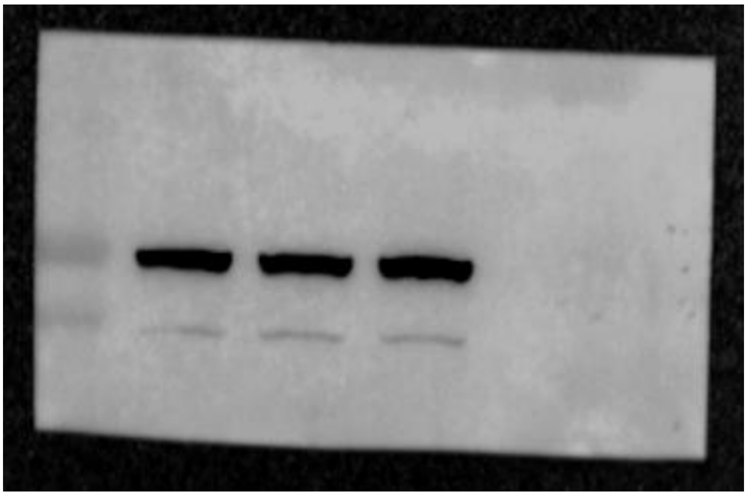

37 kDa
